# Stimulation of Toll-Like Receptors profoundly influences the titer of polyreactive antibodies in the circulation

**DOI:** 10.1038/srep15066

**Published:** 2015-10-14

**Authors:** Sreenivasulu Gunti, Ronald J. Messer, Chengfu Xu, Ming Yan, William G. Coleman, Karin E. Peterson, Kim J. Hasenkrug, Abner L. Notkins

**Affiliations:** 1 Experimental Medicine Section, Laboratory of Sensory Biology, National Institute of Dental and Craniofacial Research, National institutes of Health, Bethesda, Maryland 20892, USA; 2 Laboratory of Persistent Viral Diseases, National Institute of Allergy and Infectious Diseases, National Institutes of Health, Hamilton, Montana 59840, USA; 3 Pharmacology Section, Laboratory of Bacterial Genetics, National institutes of Diabetes and Digestive and Kidney Diseases, National Institute of Health, Bethesda, Maryland 20892, USA

## Abstract

Polyreactive antibodies are a major component of the natural antibody repertoire and bind to a variety of structurally unrelated molecules. These antibodies are thought to provide a first line of defense against bacterial infections and play a major role in the clearance of apoptotic cells. What triggers the secretion of these antibodies has remained an enigma. Using a surrogate assay for measuring polyreactive antibodies, we found that about 50% of serum IgM is polyreactive and that stimulation of TLR4^+/+^, but not TLR4^−/−^, mice resulted in a 40 fold increase in polyreactive antibodies. Stimulation of TLRs 3, 7, 9 also increased the secretion of polyreactive antibodies. Infection with a virus or tissue damage induced by a toxin similarly led to an increase in polyreactive antibodies in MyD88^+/+^, but not MyD88^−/−^ mice. We conclude that stimulation of TLRs is a key link in the mechanism of polyreactive antibody secretion into the circulation.

Natural antibodies have been known for over 100 years and are now considered part of the innate immune system. Many of these antibodies are polyreactive. That is they are capable of binding to a wide variety of structurally unrelated self (e.g., proteins, lipids, carbohydrates, DNA) and non-self (e.g., bacteria, viruses) antigens. Detailed studies on the properties of these antibodies have shown that they are of low affinity, germ-line or near germ-line in configuration and predominantly IgM, but some also are IgG or IgA^1^,^2^,^3^. Functional studies have revealed that the broad anti-bacterial activity of normal serum is largely due to polyreactive antibodies which bind to a variety of bacteria, fix complement and enhance phagocytosis^4^,^5^,^6^. In addition polyreactive antibodies can bind to cells made apoptotic by UV light or HIV infection. Upon binding to the apoptotic cells, the polyreactive antibodies also can fix complement and participate in the clearance or phagocytosis of these cells by macrophages^7^,^8^. Thus, polyreactive antibodies protect the host against both foreign invaders and its own damaged apoptotic cells before the onset of the adaptive immune response. Still other studies suggest that polyreactive antibodies may contribute to the enhancement of immune tolerance by carrying peripheral antigens to the thymus^9^ and under some circumstances polyreactive antibodies are thought to have therapeutic potential^10^,^11^.

The B cells that make these polyreactive antibodies are found in the peritoneal and pleural cavities as well as peripheral lymph nodes. In the cord blood of newborns about 50% of the B cells make polyreactive antibodies and in the peripheral circulation of adults about 15 to 20% of the B cells make polyreactive antibodies^12^,^13^,^14^. In normal serum, it is estimated that there are millions of polyreactive antibodies, many of which show different binding patterns and affinities when evaluated with a large panel of antigens. Thus, quantification of polyreactive antibodies that react with hundreds or thousands of different antigens requires a different approach than the quantification of antibodies that react with a single antigen (i.e., antigen-specific). To develop an assay for measuring polyreactive antibodies, we chose a synthetic molecule , dinitrophenol (DNP), which is not present in the environment and to which the host would not be exposed. Therefore, antibodies in the serum that react with DNP would almost certainly be polyreactive. Using two fold serial dilutions of serum, as recently described^15^, we have been able to determine the end-point or titer of polyreactive antibodies in a variety of normal and pathophysiologic states.

Unanswered, however, is what actually triggers the secretion of polyreactive antibodies that are present in normal sera. Because of the low affinity of polyreactive antibodies, it is unlikely that stimulation by one or more of the serum antigens which might bind to polyreactive B cell receptors, would provide a strong enough signal to result in the secretion of polyreactive antibodies. Rather, we hypothesized that the stimulation of Toll-Like Receptors (TLRs) on B lymphocytes, by their respective agonists, would provide strong enough stimuli to trigger the secretion of polyreactive antibodies into serum. The current study was initiated to test this possibility.

## Results

### The TLR4 agonist, lipopolysaccharide (LPS), stimulates the secretion of polyreactive antibodies into the peripheral circulation

**Figure 1:**
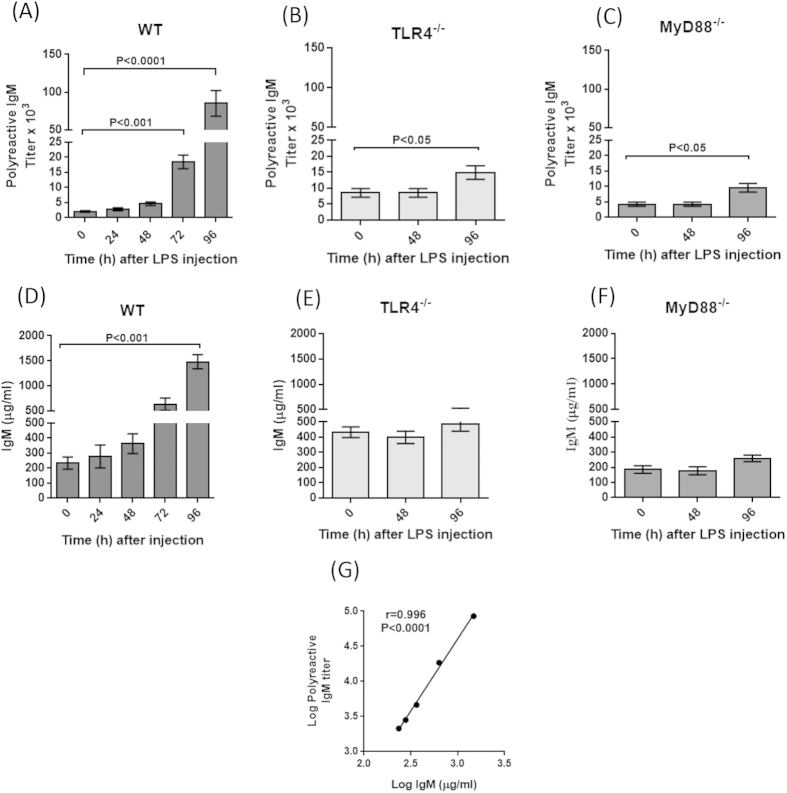
LPS stimulates polyreactive IgM antibodies. Two months old mice were injected i.p. with 5 mg/kg body weight of LPS. Titer of polyreactive antibodies in: (**A**) C57BL/6 (WT), (**B**) TLR4^−/−^ and (**C**) MyD88^−/−^ mice. Error bars represent Mean ± SEM (n = 5 mice per group, ANOVA) of three independent experiments. Sera from panels (**A–C**) first were measured for the titer of polyreactive IgM antibody and then the concentration (μg/ml) of IgM (panels (**D–F**)). Panel G shows a strong positive correlation between the titer of polyreactive IgM antibody (data from panel (**A**)) and concentration of IgM (data from panel (**D**)). The black circles in panel G represent the times at which sera were collected before and after LPS injection (0, 24, 48, 72 and 96 hours) (n = 5, Spearman r = 0.996; P < 0.001).

To determine the effect of TLRs on the secretion of polyreactive antibodies, mice were injected with LPS, a potent TLR4 ligand. As seen in Fig. 1A, the titer of polyreactive IgM antibodies in sera increased about 40 fold, from 2,100 to 85,000, at 96 hours after the injection of LPS. Proof that this increase was the result of TLR4 stimulation came from performing the same experiment in TLR4 null mice. As seen in Fig. 1B, there was only a 2 fold increase in the titer of polyreactive antibodies. Similarly, the injection of LPS in mice deficient in MyD88, which is one of the adaptor proteins required for the downstream activation of TLR signaling, also resulted in only a two fold increase in the titer of polyreactive antibodies at 96 hours (Fig. 1C). Similar findings were observed by measuring polyreactive IgG (Suppl. Fig. 1). Since it is well known that stimulation of TLR4 with LPS results in a substantial increase in serum immunoglobulins^16^, we looked for such an increase after injection of LPS. Fig. 1D shows a 6 fold increase in serum IgM concentration within 96 hours after injection of LPS, whereas no such increase was observed in TLR4 and MyD88 null mice (Fig. 1E,F). Comparing the titer of polyreactive IgM antibody (Fig. 1A) with the concentration of serum IgM at different times (i.e., 0, 24, 48, 72 and 96 hours) after LPS injection (Fig. 1D) revealed a strong positive correlation between the increase in concentration of IgM and the titer of polyreactive IgM antibodies (r = 0.996; P < 0.0001) (Fig. 1G).

### TLR9, TLR7 and TLR3 agonists increase polyreactive antibody secretion

**Figure 2:**
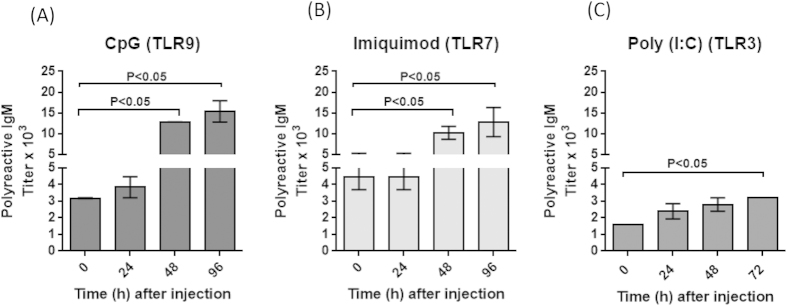
Effect of TLR9, TLR7 and TLR3 agonists on the titer of polyreactive antibodies. Two to four months old C57BL/6 mice were injected with TLR ligands (**A**) CpG; (**B**) Imiquimod; (**C**) Poly (I:C) and polyreactive antibody titers in the sera were assessed at the indicated time points. Data represents Mean ± SEM (n = 5 mice per group, ANOVA) from two independent experiments.

To determine the effect of other TLR agonists on the secretion of polyreactive antibodies, mice were injected with CpG (CpG-ODN), a TLR9 agonist, Imiquimod, a TLR7 agonist or Poly(I:C), a TLR3 agonist. Both CpG and Imiquimod produced about a fourfold increase in serum polyreactive antibodies at 96 hours after injection (Fig. 2A,B). Mice injected with Poly (I:C) showed a twofold or less increase in serum polyreactive antibodies at 72 hours after injection (Fig. 2C). As a control mice were injected with Con A, a T cell stimulant. These mice did not show any increase in the titer of polyreactive antibodies (data not shown). Thus TLRs 3, 7 and 9 had a significant, but more modest, effect on polyreactive antibody titers as compared to TLR4.

### A viral infection can trigger the secretion of polyreactive antibodies

**Figure 3:**
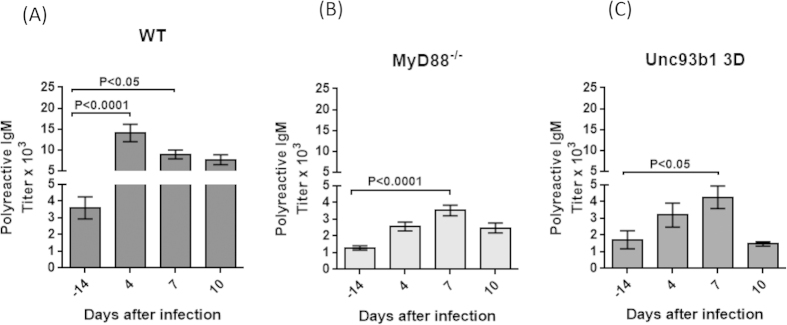
Polyreactive antibody titer in the serum increases after infection with LDV. Two months old (**A**) C57BL/6, (**B**) MyD88^−/−^ and (**C**) Unc93b13D mice were infected with LDV, blood was collected before and after infection and polyreactive IgM antibody titers in the sera were determined. Mean ± SEM (n = 10 mice per group, ANOVA).

To determine whether a viral infection could trigger the secretion of polyreactive antibodies, mice were infected with lactate dehydrogenase virus (LDV), a small RNA virus that produces up to 10^10.5^ infectious particles per ml of serum within 24 hours after infection^17^. It was previously shown that innate immune response to LDV infection was dependent on TLR7 signaling^18^. Consistent with the results from the TLR7 ligand imiquimod (Fig. 2B), the polyreactive antibody titer in the serum of LDV-infected mice increased from about 3,500 at 14 days before infection to nearly 15,000 at 4 days after infection and then decreased (Fig. 3A). In contrast, the titer of polyreactive antibody in MyD88 null mice at 14 days before infection was only 1,500 and increased to about 3,500 at 7 days after infection (Fig. 3B). Similarly, in Unc93b13D mutant mice, in which a single nucleotide mutation in Unc93b1 results in deficient TLR3, TLR7 and TLR9 responses^19^, the titer of polyreactive antibodies at 14 days before infection was about 1,800 and increased to 4,000 at 7 days after infection (Fig. 3C). These experiments show that a viral infection can increase the titer of polyreactive antibodies and that the effect is largely dependent on TLR signaling. Interestingly, the low homeostatic titer of polyreactive antibodies in both MyD88 null mice and Unc93b13D mutant mice, as compared to wild type mice, also suggests a role for TLRs in the maintenance of serum polyreactive antibodies.

### Carbon tetrachloride (CCl_4_) induced liver damage triggers the secretion of polyreactive antibodies

**Figure 4:**
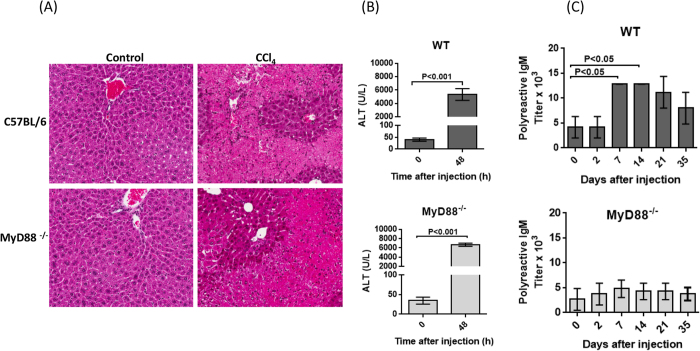
CCl_4_ induced liver damage enhances the secretion polyreactive antibodies. Two months old mice (n = 5) were injected with 2 ml/kg body weight of CCl_4_ i.p. (**A**) Histopathology and (Original magnification X10) (**B**) elevated serum ALT levels show the induction of liver damage (Mann-Whitney U test) in both C57BL/6 wild type and MyD88 null mice. (**C**) Polyreactive antibody titer increases after injection of CCl_4_ in wild type mice, but not MyD88 null mice. Mean ± SEM (n = 5 mice per group, ANOVA).

Tissue injury is associated with the cellular release of damage-associated molecular pattern molecules such as heat shock proteins and DNA. These molecules can act through TLRs to initiate innate immune responses and inflammation^20^. To study the effect of tissue damage on polyreactive antibody levels in the serum, C57BL/6 mice were given a single sub-lethal dose of CCl_4_ and polyreactive antibodies were measured at different times thereafter. Histology clearly shows the damage to liver cells in CCl_4_ injected mice (Fig. 4A). Elevated serum alanine amino transferase (ALT) levels in CCl_4_ injected mice (Fig. 4B) confirmed the effective induction of liver damage. A threefold increase in the titer of polyreactive antibodies was observed at 7 and 14 days after CCl_4_ injection (Fig. 4C). To determine if this effect was due to TLR signaling, MyD88 null mice were injected with CCl_4._ The degree of histopathology and the elevation of serum ALT were similar to that observed in the C57 BL/6 mice (Fig. 4A,B, lower panels). However, there was no increase in the titer of polyreactive antibodies in the serum of MyD88 null mice (Fig. 4C, lower panel). This strongly suggests that the stimulation of MyD88- dependent TLRs by endogenous agonists is involved in the secretion of polyreactive antibodies.

### High percentage of natural IgM is polyreactive

Purified mouse IgM was passed through DNP or control BSA columns and the amount of IgM that bound was determined. Our studies showed that 40 to 60% of the IgM bound to the DNP columns whereas only 5 to 15% bound to the BSA columns. As seen in Supplemental Fig. 2, the IgM eluted from the DNP columns readily bound a number of different antigens characteristic of polyreactive antibodies, whereas an equal amount of IgM eluted from the BSA columns showed little or no reactivity with the same panel of antigens.

## Discussion

What stimulates the secretion of polyreactive antibodies and maintains a rather constant level in the circulation has remained an enigma. The work reported here shows that TLRs play a critical role in the secretion of polyreactive antibodies. Stimulation of TLR4 with its agonist, LPS, resulted in nearly a 40-fold increase in polyreactive antibodies within 96 hours. The same experiment performed in TLR4 or MyD88 null mice resulted in only a twofold increase in polyreactive antibodies providing strong evidence that the increase of polyreactive antibodies in the WT mice was largely TLR-mediated and not BCR-mediated. However, almost certainly, some antigens may act through both the TLR and BCR.

TLRs are expressed on all B cells and the stimulation of these receptors increases the secretion of antibodies. The reason, however, for the massive increase in polyreactive antibodies after stimulation of TLR4 is due to the fact that about 15 to 20% of the B cells in the peripheral circulation can produce polyreactive antibodies and the total percentage of these cells is even higher when the polyreactive B cells in the various body compartments (e.g., peritoneal and pleural cavities, bone marrow, lymph nodes and spleen) are included^12^,^13^,^14^. Of particular interest, is the observation (Fig. 1G) that following stimulation of TLR4 with LPS, there is a strong positive correlation between the titer of polyreactive antibodies and the concentration of IgM (r = 0.99, P = 0.0001). However, this increase in polyreactive antibodies (about 40 fold) is disproportionately higher than the increase in IgM (6 fold). This disproportionateness is best explained by the high percentage of B cells in the repertoire that can secrete polyreactive antibodies.

The demonstration that TLRs agonists significantly increase the level of polyreactive antibodies suggested that infectious agents might contain and tissue damage might lead to the release of TLR agonists and thereby increase the secretion of polyreactive antibodies. Both infection with LDV and liver damage caused by CCl_4_ did just that. The fact that stimulation of TLRs was responsible for this increase in polyreactive antibodies was demonstrated by performing the same experiments with MyD88 null mice which showed a marked decrease in the secretion of polyreactive antibodies. It is estimated that under normal conditions, a billion cells a day might undergo apoptosis resulting in the release of a variety of cellular factors some of which are TLR agonists^21^,^22^. This could explain the constant presence of polyreactive antibodies in normal serum.

There is little information, however, on the actual percentage of polyreactive IgM in the total IgM repertoire. By use of the DNP surrogate assay we have been able to obtain a rough estimate. Our experiments showed that about 50% of the serum IgM repertoire is made up of polyreactive antibodies (Suppl. Fig. 2). Although DNP appears to be the best surrogate of the many that we have tested (unpublished data), our finding of 50% polyreactivity in the IgM repertoire may actually be an under estimate because some polyreactive antibodies may not bind well to DNP because of very low affinity^23^.

The demonstration of the importance of TLR in the secretion of polyreactive antibodies and the DNP surrogate assay to measure polyreactive antibodies opens a new avenue to study the role of not only polyreactive IgM, but also polyreactive IgG and IgA, under both normal and pathophysiologic conditions. Although not extensively studied, serum from mice treated with LPS was tested for polyreactive IgG. As seen in Suppl. Fig. 1, there was about a 10 fold increase in polyreactive IgG as compared to a 40 fold increase in polyreactive IgM (Fig. 1). No increase in polyreactive IgG was found in the MyD88 and TLR4 null mice. This is consistent with our earlier observations, using hybridoma technology, which showed that the frequency of monoclonal polyreactive IgG antibodies was considerably less in mice than monoclonal polyreactive IgM antibodies^24^.

In the experiments described here, the titer of polyreactive antibodies was measured primarily after an acute event (i.e., injection of TLR agonists, infection with a virus or tissue damage induced by a toxin). In the future, it will be particularly interesting to evaluate the effect of chronic diseases on the titer of polyreactive antibodies in the serum of mice and humans. The high frequency of IgM antibodies that are polyreactive points to the importance of these antibodies in innate immunity and suggests that some of these antibodies may actually be precursors of high affinity antibodies found in the adaptive immune system^25^,^26^. Although largely ignored in the past because of their low affinity, the demonstration that as much as half of the IgM repertoire is polyreactive, and that these antibodies can bind to and enhance the phagocytosis of bacteria and apoptotic cells, sheds new light on the importance of polyreactive antibodies.

## Materials and Methods

### Surrogate assay for determining the titer of polyreactive antibody

In most studies, the polyreactivity of a monoclonal polyreactive antibody is assessed using a standard panel of antigens. An antibody molecule is said to be polyreactive based on its ability to bind to that panel of antigens. However, measuring polyreactive antibodies quantitatively in the serum is complicated as there are millions of polyreactive antibodies in the serum, with different affinities and binding patterns. In the present paper, we developed a surrogate assay (an index) for measuring polyreactive antibodies in the serum. First we compared the binding of a monoclonal polyreactive antibody with a panel of standard antigens used in polyreactive antibody assays such as beta-galactosidase (B-gal), single stranded DNA (ssDNA), insulin etc., and in addition with a number of synthetic molecules including dinitrophenol, trinitrophenol, azobenzenearsonate, 4-hydroxy-3-iodo-5-nitrylphenyl acetyl (NIP) etc. Then we tested these synthetic molecules with sera from normal mice (n = 20) (Suppl. Fig. 3). Normal mouse sera reacted with these molecules as did sera from different strains of mice (Gunti and Notkins, 2015,) and as did sera following injection of mice with TLR ligands (e.g. LPS, Suppl. Fig. 4). In all these cases, the sera that reacted with the panel of standard antigens also reacted with the synthetic molecules and most consistently with DNP. Therefore, we decided to use the DNP assay as a surrogate for polyreactivity. DNP is not in the environment and individuals are not exposed to it. Therefore antibodies reacting with DNP almost certainly would have to be polyreactive. The value of the surrogate assay is that it provides an index (a single number) that can be used to compare one experiment with another and to compare results from different laboratories.

The end point dilution method was used to determine the titer of polyreactive antibodies by ELISA. Microtiter 96 well plates (Pierce, Rockford, IL, USA) were coated with 100 μl of 5 μg/ml of 2,4-dinitrophenol-bovine serum albumin (DNP-BSA) (Bioresearch Technologies, Novato, CA, USA) in 50 mM carbonate buffer pH 9.6, overnight at 4°C. The plates were washed three times with phosphate buffered saline containing 0.05% Tween-20 (PBST) and blocked with 200 μl of PBS containing 1% bovine serum albumin for two hours at room temperature. Following blocking, 100 μl of two fold serially diluted serum samples were added to the wells and incubated at room temperature for one hour. Plates were then washed and bound antibodies were revealed by goat anti-mouse IgM labeled with Biotin or goat anti-mouse IgG labeled with horse radish peroxidase (both from Southern Biotech, Birmingham, AL, USA). Subsequently, plates were washed and developed with ABTS substrate (Invitrogen, Carlsbad, CA, USA) at room temperature for 15 min and the reaction was stopped with 100 μl of 0.1 M citric acid containing 0.01% sodium azide. Optical density (O.D.) was measured at 405 nm in a micro-plate reader (Molecular Devices, Sunnyvale, CA, USA)). To determine the background, sera from 20 normal C57BL/6 mice were analyzed with appropriate controls. Under the conditions of the assay, the background was about 0.08 O.D and an O.D. of 0.2 was set as the cut-off point. Polyreactive antibody titers were defined as the reciprocal of the highest serum dilution that produced an O.D value above the cutoff.

### Quantification of serum immunoglobulins

IgM and IgG concentrations in the sera were determined by standard sandwich ELISA. Serum IgM and IgG were captured by coating the ELISA plates with goat anti-mouse IgM or goat anti-mouse IgG respectively. Bound IgM and IgG were revealed by HRP labeled rat anti-mouse IgM and goat anti-mouse IgG respectively.

### Injection of mice with LPS and other TLR ligands

Groups of five C57BL/6, TLR4^−/−^ and MyD88^−/−^ mice (both on C57BL/6 background), two to four months of age, were injected i.p. with LPS at 5 mg/kg body weight dosage (*E. coli O55:B5*, Sigma, St. Louis, MO, USA) dissolved in endotoxin free PBS. Blood was collected at different times before and after injection and serum was stored at −80 °C until use. Polyreactive antibodies against different antigens and total Igs were measured by ELISA. Phosphorothioate CpG oligonucleotide 5′-GAGAA**CG**CT**CG**ACCTTCCAT-3′ and control oligonucleotide 5′-GAGAA**GC**CT**GC**ACCTTCCAT-3′ were synthesized at the Core Facility of Center for Biological Evaluation and Research, Food and Drug Administration, Bethesda, USA and injected i.p at a dose of 500 μg per mouse. Imiquimod (R837) and PolyI:C (In Vivogen, San Diego, CA, USA) were dissolved in sterile endotoxin free PBS and injected i.p at a dose of 250 and 100 μg/mouse, respectively. Polyreactive antibody at the indicated time points was measured by ELISA.

### Infection of mice with Lactate Dehydrogenase-elevating Virus (LDV)

Mice used in the experiments were all females, 13–22 weeks of age. C57BL/6 mice were obtained from Jackson Laboratories. MyD88^−/−^ mice^27^ and the Unc93b13D mice^19^, both on C57BL/6 background, were bred at the NIAID, Rocky Mountain Laboratories. The LDV stock was prepared as previously described^28^. Mice were infected i.p. at a 1/100 dilution of virus stock containing approximately 10^7^ ID_50_ of LDV and blood was collected at the times indicated for polyreactive antibody measurement.

### Induction of liver damage by carbon tetrachloride (CCl_4_)

Two to four months old C57BL/6 and MyD88^−/−^ mice were injected i.p. with CCl_4_, 2 ml/kg body weight (Sigma). To assess liver damage, a group of mice were sacrificed two days after CCl_4_ injection, liver was dissected, fixed in formaldehyde and sections were stained with Hematoxyline and Eosin. Images were captured using Leica Scanscope (Aperio CS2) (Leica, Heidelberg, Germany). Serum alanine amino transferase (ALT) was measured at the NIH Clinical Center Pathology Laboratory. After confirming the liver damage by elevation of ALT, mice were bled at the times indicated after CCl_4_ injection and polyreactive antibody titers determined.

### Quantification of polyreactive antibodies in serum IgM

To determine the percentage of polyreactive antibodies in the serum, 5 mg of DNP-BSA was coupled to 1 ml NHP-activated Sepharose columns (GE Healthcare, Piscataway, NJ, USA) per manufacturer’s instructions. Different concentrations of mouse IgM, purified from plasma (>95% pure, Rockland Immunochemicals, Gilbertsville, PA, USA) was applied to the DNP columns at a flow rate of 0.5 ml/min. The percentage of IgM that bound to DNP was calculated from the quantity of IgM (μg) loaded on to the DNP columns minus the quantity of IgM in the flow through and wash fractions as determined by ELISA. A similar experiment was conducted on sepharose columns coupled with BSA. To assess the polyreactivity of each fraction from both DNP and BSA columns, an equal amount of IgM from each fraction was tested for their reactivity against the panel of antigens by ELISA.

All animal experiments were performed in accordance with animal study protocols approved by NIDCR, NIDDK and the NIAID Rocky Mountain Laboratories Institutional Animal Care and Users Committees.

### Data analysis

Data are presented as Mean ± the Standard Error of the Mean (SEM) and differences between groups, unless otherwise specified, was analyzed by one way analysis of variance (ANOVA) followed by Kruskal-Wallis multiple comparisons using Graphad Prism 5 software. A *P* value ≤ 0.05 was considered significant.

## Additional Information

**How to cite this article**: Gunti, S. *et al.* Stimulation of Toll-Like Receptors profoundly influences the titer of polyreactive antibodies in the circulation. *Sci. Rep.* **5**, 15066; doi: 10.1038/srep15066 (2015).

## Supplementary Material

Supplementary Information
