# Supplementary material for: Stimulation of Toll-Like Receptors profoundly influences the titer of polyreactive antibodies in the circulation
Source: Sci Rep. 2015 Oct 14;5:15066. doi: 10.1038/srep15066 (PMC4604466; doi:10.1038/srep15066)
Supplement: Supplementary Information [file srep15066-s1.pdf]

Stimulation of Toll-Like Receptors profoundly influences the titer of polyreactive antibodies in the circulation

Sreenivasulu Gunti[1], Ronald J. Messer[2], Chengfu Xu[3], Ming Yan[3], William G. Coleman Jr.[3,4,] Karin E. Peterson[2], Kim J. Hasenkrug[2] and Abner L. Notkins[1*]

Legend for Supplemental Figures

Suppl. Fig. 1. LPS stimulates the secretion of polyreactive IgG antibodies: Two months old (A) C57BL6, (B) TLR4$^{-/-}$ and (C) MyD88$^{-/-}$ mice were injected with LPS and titer of polyreactive IgG antibody was determined by ELISA on the same sera as used in Fig.1. Mean ±SEM (n=5 mice per group, ANOVA) from two independent experiments.

Suppl. Fig.2. Eluted polyreactive antibodies: Purified mouse IgM was loaded on to (A) DNP columns or (B) BSA control columns and the percentage that bound was calculated. Equal amounts of IgM from the eluted fractions then were tested for reactivity with different antigens. Data represents Mean +/- SEM of two independent experiments (ANOVA).

Suppl.Fig.3. Surrogate assay for measuring polyreactive antibody titer: (A) ELISA plates were coated with different antigens and polyreactive antibody titer was determined by serial two fold dilutions. Dotted line represents plate background activity. (B) Polyreactive antibody titer with different antigens. Data represents Mean ±SEM (n=20 mice).

Suppl.Fig.4. LPS triggers polyreactive antibody secretion. The polyreactive antibody titer in sera after injection of mice with LPS. Elisa plates were coated with (A) Beta-galactosidase, (B) single stranded DNA and (C) NIP. Data represents Mean +/- SEM of (n=5 mice per group) two independent experiments (ANOVA).

Suppl. Fig. 1.

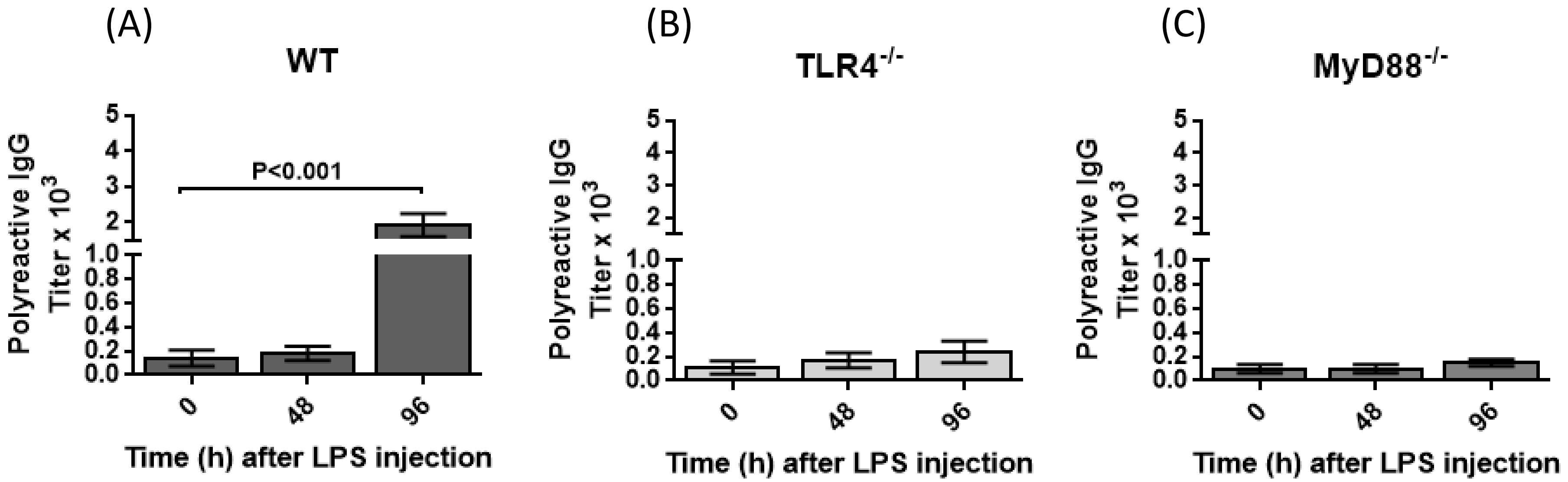

(A)
WT
Polyreactive IgG Titer x 10^3
5
4
3
2
1.0
0.8
0.6
0.4
0.2
0.0
P<0.001
0
48
96
Time (h) after LPS injection
(B)
TLR4-/-
Polyreactive IgG Titer x 10^3
Time (h) after LPS injection
(C)
MyD88-/-
Polyreactive IgG Titer x 10^3
Time (h) after LPS injection

Suppl. Fig. 2.

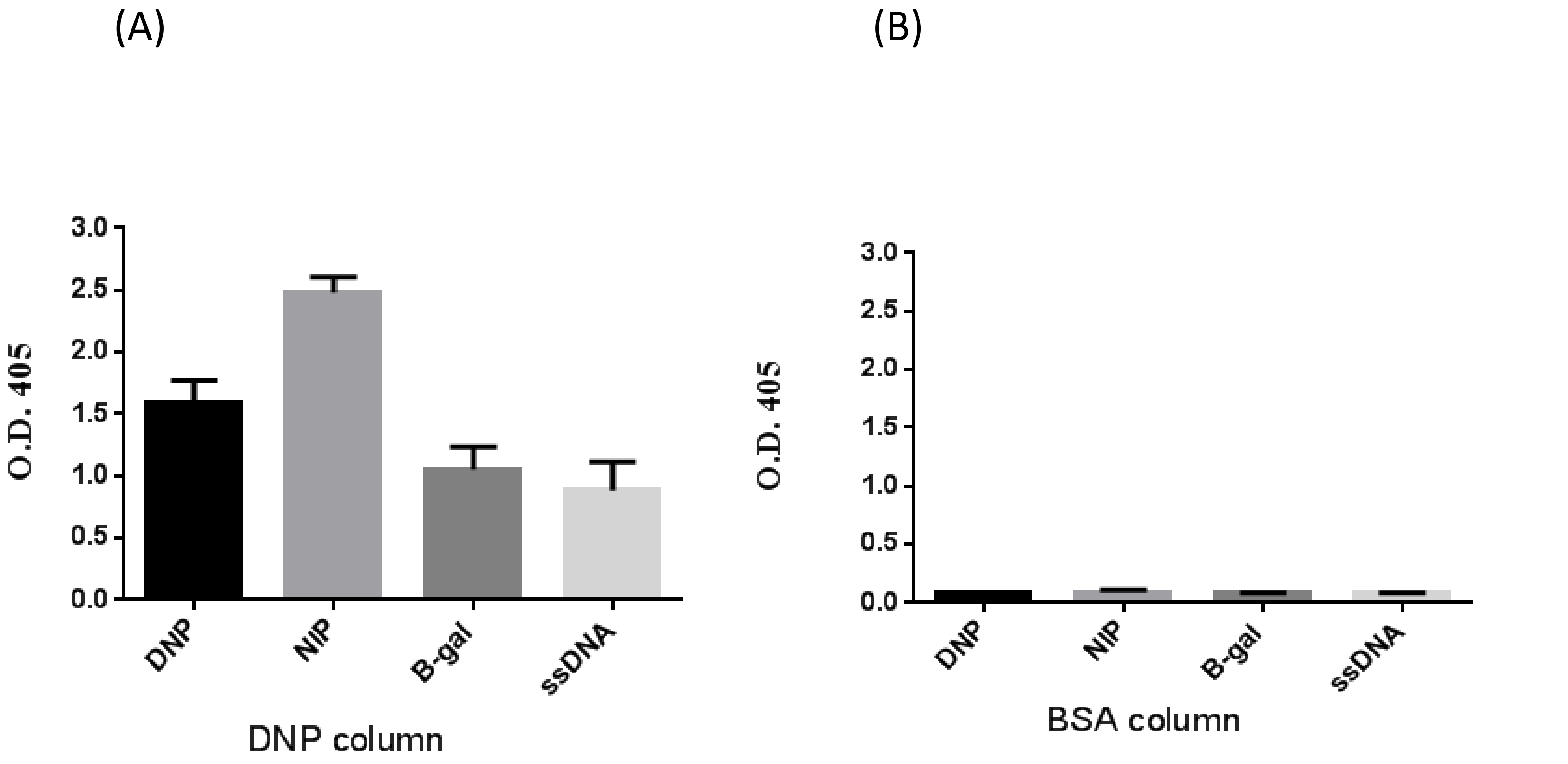
(A)
(B)
3.0
2.5
2.0
1.5
1.0
0.5
0.0
O.D. 405
DNP
NIP
B-gal
ssDNA
DNP column
BSA column

Suppl. Fig. 3.

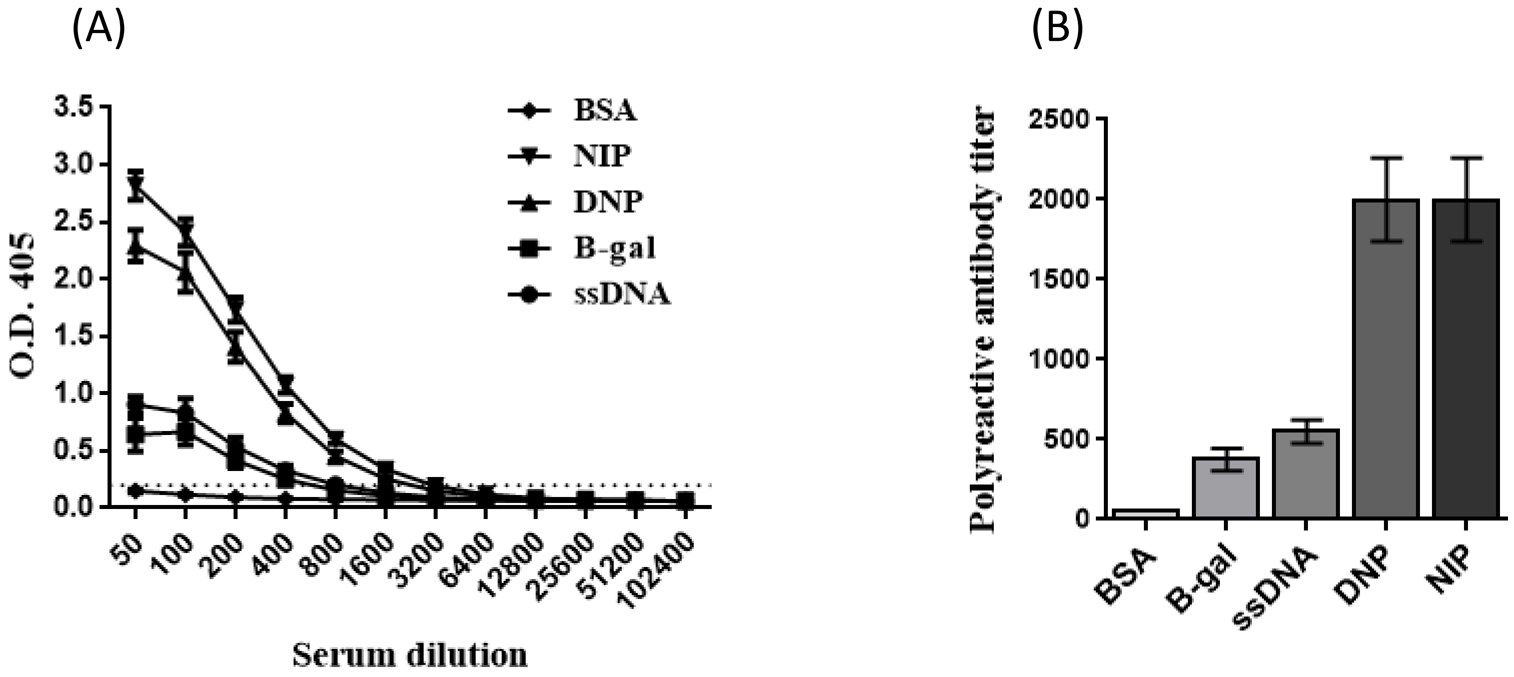
(A)
(B)
O.D. 405
3.5
3.0
2.5
2.0
1.5
1.0
0.5
0.0
BSA
NIP
DNP
B-gal
ssDNA
50
100
200
400
800
1600
3200
6400
12800
25600
51200
102400
Serum dilution
Polyreactive antibody titer
2500
2000
1500
1000
500
0
BSA
B-gal
ssDNA
DNP
NIP

Suppl. Fig. 4.

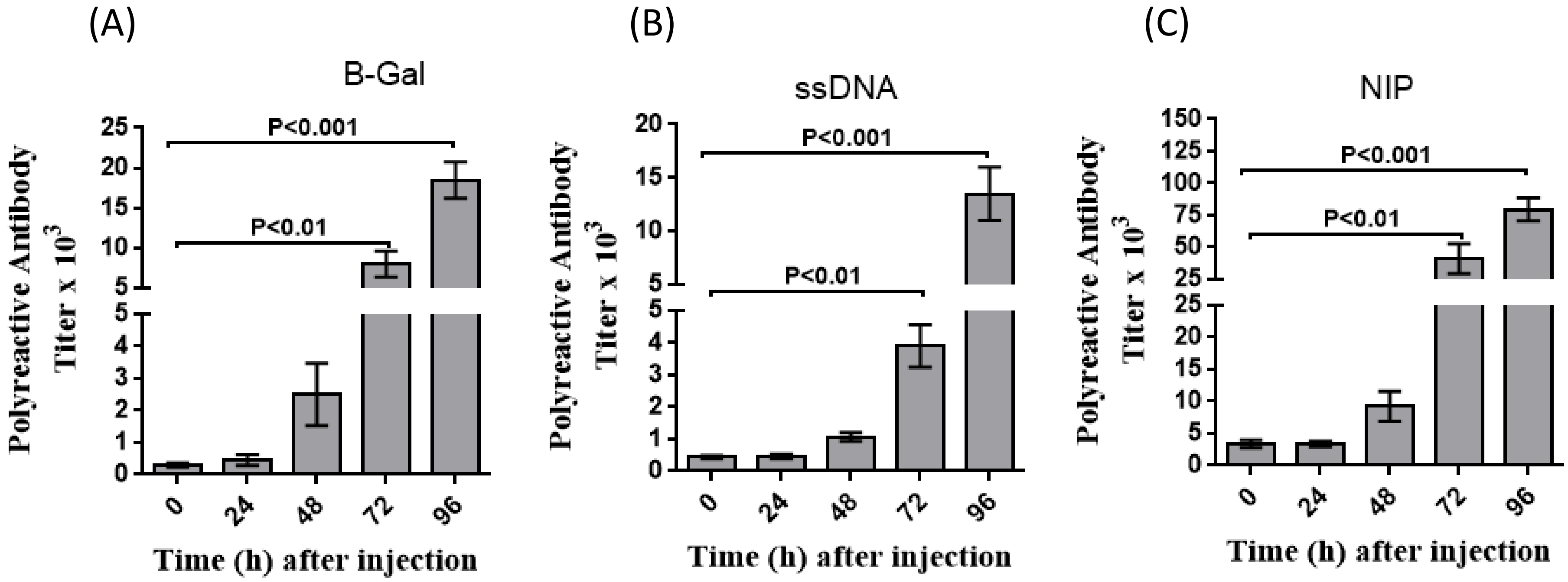
(A)
B-Gal
Polyreactive Antibody Titer x 10^3
P<0.001
P<0.01
25
20
15
10
5
5
4
3
2
1
0
0
24
48
72
96
Time (h) after injection
(B)
ssDNA
Polyreactive Antibody Titer x 10^3
P<0.001
P<0.01
20
15
10
5
5
4
3
2
1
0
0
24
48
72
96
Time (h) after injection
(C)
NIP
Polyreactive Antibody Titer x 10^3
P<0.001
P<0.01
150
125
100
75
50
25
25
20
15
10
5
0
0
24
48
72
96
Time (h) after injection
